# Proximal Anchoring Technique: A Novel Technique to Prevent Coronary Stent Longitudinal Deformation

**DOI:** 10.31083/RCM44250

**Published:** 2026-02-10

**Authors:** Song Zhang, Yongtai Gong, Yiqun Zhang, Danghui Sun, Yue Li

**Affiliations:** ^1^Department of Cardiology, The First Affiliated Hospital, Harbin Medical University, 150001 Harbin, Heilongjiang, China; ^2^The Vivolight Medical Device & Technology Co., Ltd., 518000 Shenzhen, Guangdong, China; ^3^NHC Key Laboratory of Cell Translation, Harbin Medical University, 150001 Harbin, Heilongjiang, China; ^4^Key Laboratory of Hepatosplenic Surgery, Harbin Medical University, Ministry of Education, 150001 Harbin, Heilongjiang, China; ^5^Key Laboratory of Cardiac Diseases and Heart Failure, Harbin Medical University, 150001 Harbin, Heilongjiang, China; ^6^Heilongjiang Key Laboratory for Metabolic Disorder & Cancer Related Cardiovascular Diseases, 150081 Harbin, Heilongjiang, China

**Keywords:** stent elongation and shortening, post-dilation, percutaneous coronary intervention

## Abstract

**Background:**

Precise coronary stent implantation is crucial for ostial and partial bifurcation lesions during percutaneous coronary intervention (PCI). Conventional post-dilation coronary stent implantation often causes longitudinal stent deformation (LSD); meanwhile, even a small area of protrusion into the proximal main branch (MB) can lead to severe problems. This study aimed to introduce a novel post-dilation technique, the proximal anchoring technique (PAT), and evaluate the associated feasibility and efficacy in achieving precise stent implantation and preventing LSD.

**Methods:**

This bench study was performed in a tapered silicon vessel model, in which 3.5 × 28 mm-sized everolimus-eluting stents (Xience Xpedition™; Abbot, USA) were deployed at a nominal pressure. Post-dilation was conducted using two different strategies: the proximal anchor followed by distal post-dilation group (PAT group) and the conventional post-dilation group (dilation from distal to proximal) (D-P group). After each step, the subsequent changes in stent length were measured by optical coherence tomography (OCT). Additionally, three clinical PCI cases in which PAT and conventional post-dilation were employed are presented.

**Results:**

The longitudinal elongation of stents was significantly increased in the D-P group compared with the PAT group (*p* < 0.001). The OCT measurements showed that the stents were elongated during every step of the procedure in the D-P group (29.35 ± 0.10 mm vs. 29.65 ± 0.10 mm; *p* = 0.0054), but only slightly elongated in the first step of the post-dilation in the PAT group (28.73 ± 0.12 mm vs. 28.87 ± 0.12 mm; *p* = 0.2262).

**Conclusions:**

We present a novel technique, PAT, to assist in more precise coronary stent implantation by preventing LSD for partial ostial and bifurcation lesions during PCI.

## 1. Introduction

During percutaneous coronary interventions (PCI), complex coronary lesions, including aortic ostial lesions and partial bifurcation lesions (such as Medina 0,1,0 and 0,1,1 and 0,0,1), require precise stent deployment [1, 2]. Currently, there are several techniques to help accurate stent placement in the ostium including multiple angiographic views assist, the aorta flowing wire technique, the Szabo technique (anchor wire), and intra-luminal imaging assist [3, 4, 5]. However, the low accuracy, complex operation steps, high economic costs and increased complications have limited the clinical application of these methods [6].

Longitudinal stent deformation (LSD) is a relatively common complication during PCI procedures, which is defined as the distortion of a stent in the longitudinal axis [7, 8, 9, 10]. Although LSD is mainly described as focal or overall longitudinal stent shortening, recent studies have shown that longitudinal stent elongation was over four times more common than stent shortening in clinical practice [11, 12]. During aorta ostial lesion interventions, longitudinal stent elongation causes strut damage induced by the guiding catheter or the post-dilation balloon. This becomes a potential nidus for thrombus formation, plus difficulties with ostial catheter engagement and wire advancement during future catheterizations. Stents that are implanted too proximally in the side branch (SB) could cause struts to protrude into the main branch (MB), which may complicate future revascularization or lead to episodes of acute thrombosis. The problem of unintentional stent elongation and the accurate location of the proximal stent edge during PCI remains unresolved.

In this report, we introduce the proximal anchoring technique (PAT), which consists of the proximal portion of the stent with a non-compliant balloon size ratio 1:1 with the proximal reference vessel followed by distal sequential post-dilation. We evaluate the safety and effectiveness of the PAT in a series of clinical PCI cases, to achieve precise stent implantation and prevent the conventional post-dilation induced LSD.

## 2. Methods

### 2.1 Description of Bench Testing

Bench testing was performed for the PAT in a three dimensional (3D) printed, double-sided polished, pure saline-filled silicone tapered vessel phantoms (Shonankasei Co., Kanagawa, Japan). The procedures were performed by operators who had sufficient practical (human and bench) experience with PCI. Although clinically drug eluting stents (DES) and bioabsorbable scaffolding stents (BVS) are associated with more fractures than bare metal stents, the drug coating does not alter the potential for fracture of a stent platform on the bench [13, 14]. The Xience Xpedition™ everolimus-eluting coronary stent (Abbott, Abbott Park, IL, USA) was used for the experiments. The distal carina site of the vertical side branch was used as a marker to calculate the proximal length of stent elongation. The exact parameters of the tapered vessel phantom are shown in **Supplementary Fig. 1**.

After the PAT, optical coherence tomography (OCT) imaging was systematically performed to evaluate the relative length of stent elongation [15]. Pullback runs were performed at 20 mm/s with a Dragonfly™ Duo OCT catheter (Abbott Vascular, Temecula, CA, USA) and analysis was performed using a dedicated workstation (C7-XR™ OCT intravascular imaging system; Abbott Vascular). Images were recorded at 100 frames per second. The measurement parameters of the tapered vessel phantom by OCT are shown in **Supplementary Fig. 2**.

### 2.2 Technical Description

**Fig. 1.**
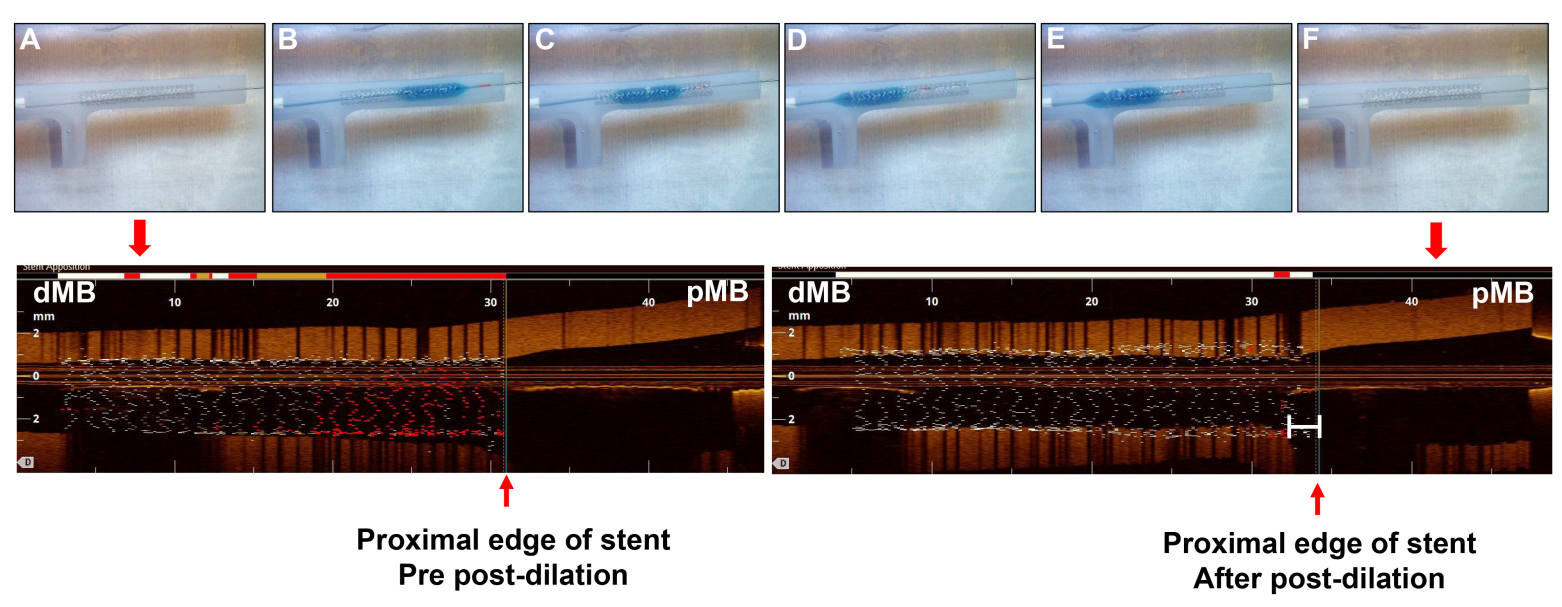
**Study procedure of post-dilation from distal to proximal direction**. Upper panel: (A) A 3.5 × 28 mm sized stent with precise placement, and the proximal stent edge located at the distal of carina of main branch. (B–D) A 3.5 mm × 12 mm non-compliant balloon sequentially post-dilated in the stent at 12 atm, 15 atm and 18 atm. The distal or proximal radiopaque marker of the post-balloon located at the edge of the stent, respectively. (E) A 4.0 mm × 12 mm non-compliant balloon post-dilated in the stent at 20 atm. (F) The picture of stent after post-dilation. Lower panel: The corresponding OCT view of the stent in bench testing. OCT, optical coherence tomography; dMB, distal main branch; pMB, proximal main branch.

**Fig. 2.**
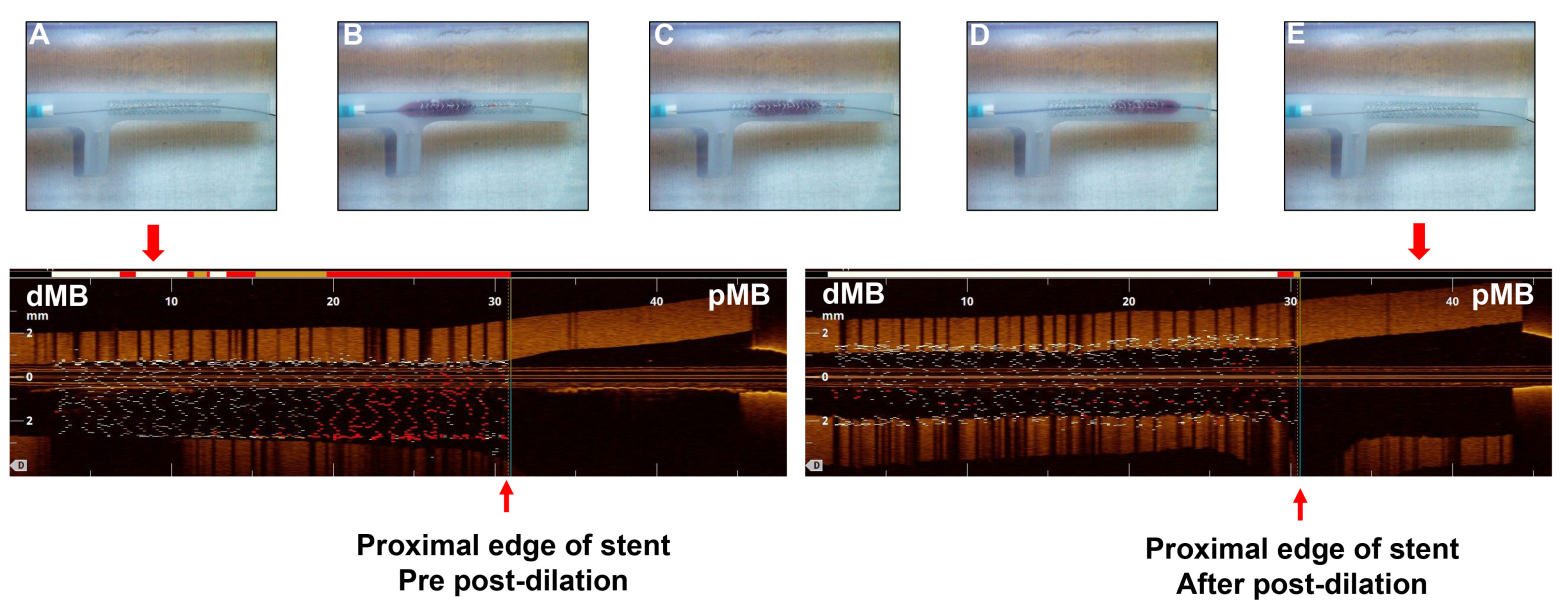
**Study procedure of post-dilation from proximal to distal direction**. Upper panel: (A) A 3.5 × 28 mm sized stent with precise placement, and the proximal stent edge located at the distal of carina of main branch. (B) A 4.0 mm × 12 mm non-compliant balloon post-dilated in the stent at 20 atm. The proximal radiopaque marker of the post-balloon is located at the proximal edge of the stent. (C,D) A 3.5 mm × 12 mm non-compliant balloon sequentially post-dilated in the stent at 12 atm and 15 atm. (E) The picture of stent after post-dilation. Lower panel: The corresponding OCT view of the stent in bench test.

PAT was investigated both in a bench model and clinically in patients undergoing PCI, with stents implanted precisely, followed by post-dilation of PAT. In this *in vitro* model, a 3.5 × 28 mm sized stent was deployed at a nominal pressure (10 atm) for 20 s in a tapered fashion. Then, we performed post-dilation by using two methods: the D-P group (Fig. 1), with post-dilation ballooning from the distal to proximal direction, and the PAT group (Fig. 2), with post-dilation ballooning from the proximal to distal site of the stent. For the PAT group, the size of the larger post-dilation balloon should be determined by 1:1 ratio with the diameter of the proximal reference vessel.

#### 2.2.1 Distal–Proximal Post-Dilation Demonstration (D-P Group)

In this group, after the precise stent placement at the ostium was performed, the stent length and malposition were measured by OCT scans. Next, post-dilation was performed from the distal to proximal stent at 12 atm, 15 atm, and 18 atm respectively using a 3.5 mm × 12 mm non-compliant balloon (Sprinter®, Medtronic Co., Minneapolis, MN, USA). Then, the balloon size was increased, post-dilation was conducted at the proximal edge of the stent using a 4.0 mm × 12 mm non-compliant balloon (Sprinter®, Medtronic Co., USA) at 20 atm.

#### 2.2.2 Proximal–Distal Post-Dilation Demonstration (PAT Group) 

In this group, after the precise stent placement at the ostium was performed, the stent length and malposition were measured by OCT scans. Post-dilation was performed from the proximal to distal direction. First, post-dilation was performed at the proximal edge of the stent by using a 4.0 mm × 12 mm non-compliant balloon (Sprinter®, Medtronic Co., USA) at 20 atm. Then, the balloon size was decreased, post-dilation was conducted in the middle and distal of the stent using a 3.5 mm × 12 mm non-compliant balloon (Sprinter®, Medtronic Co., USA) at 15 atm and 12 atm, respectively.

Every step of post-dilation was performed for 20 s. Each group’s bench test will undergo 3-4 repeated operations to ensure stable results. At the end of the procedure, the OCT scan was repeated to measure the stent length and to determine if there was any malposition.

### 2.3 Statistical Analysis

The distribution of continuous variables was examined using the Shapiro–Wilk test. The paired Student’s *t*-test was used to compare the stent length before and after stent post-dilation during each step of the procedure. Student’s *t*-test was used to compare the differences in the percentage change of stent length during post-dilation between the D-P and PAT groups. All calculations were performed using R software version 4.3.0 (https://www.r-project.org/) and Figures were drawn using GraphPad Prism version 10.1.2 (GraphPad Software LLC, San Diego, CA, USA). A *p*-value of <0.05 was considered statistically significant.

## 3. Results 

**Fig. 3.**
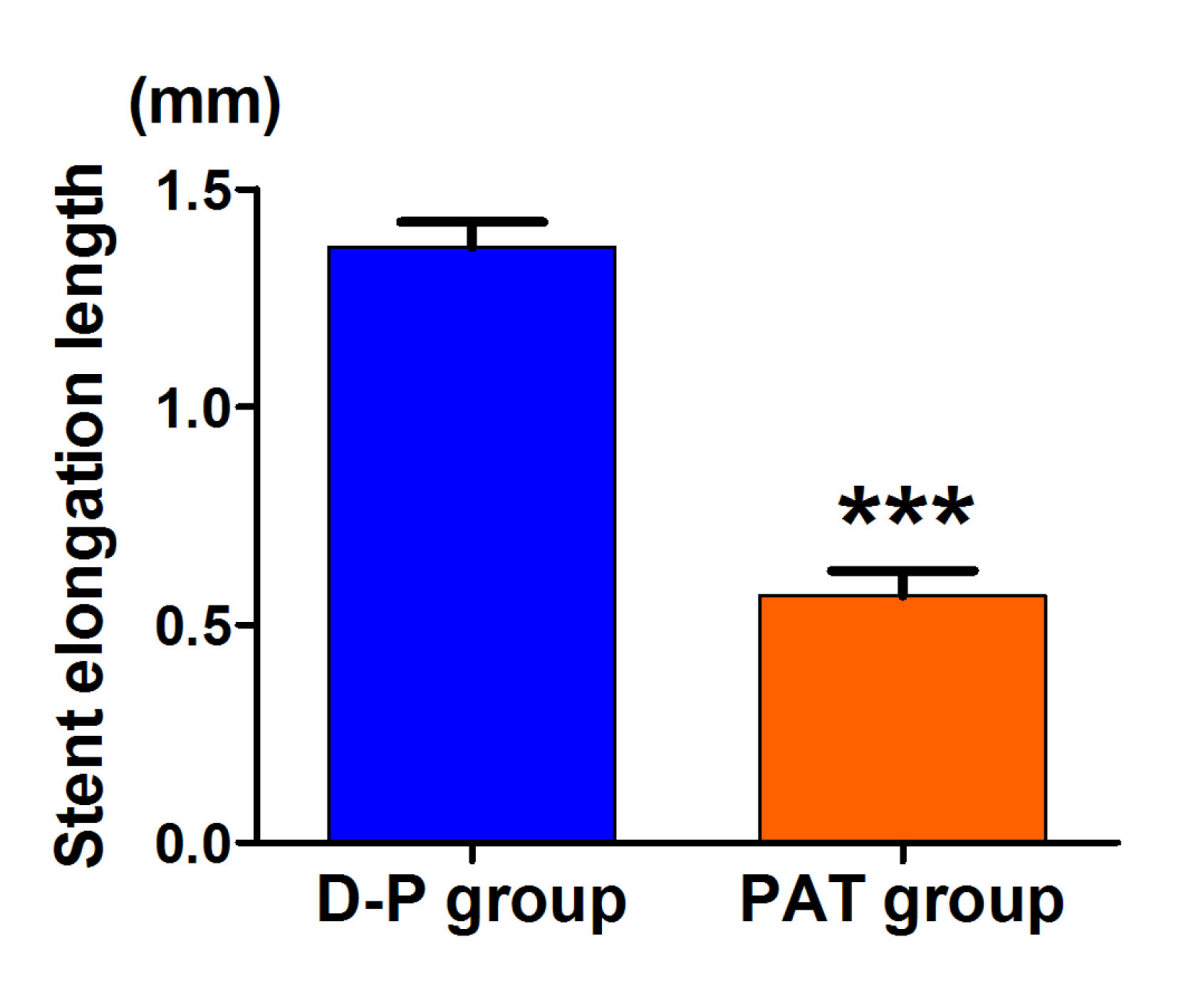
**Stent elongation length in the PAT group and conventional post-dilation group**. ***p < 0.001 vs. D-P group. Data are expressed as the mean ± SEM. SEM, standard error of the mean.

In the bench test, there were no differences in stent length between two groups immediately after stent deployment. In the D-P group, the stents were significantly elongated during the first and second post-dilations. However, in the PAT group, the stents were slightly elongated during the first post-dilation, whereas it was not significantly elongated during the second post-dilation (Table 1). In addition, the stent length after first and second post-dilation of PAT group were significantly shorter than that in D-P group (Table 1). Compared to the D-P group, the distance of the stent length proximally was significantly reduced in the PAT group after the first post-dilation, and the elongated distance was not statistically different after the second post-dilation between the two groups (Table 2). The total elongated distance was significantly shorter in PAT group than that in D-P group (Fig. 3).

**Table 1.** **The measurements of stent length at the end of each step**.

	Nominal stent length (mm)	Immediately after stent deployment (mm)	*p* value (Nominal vs. Immediately)	After 1st post-dilation (mm)	*p* value (Immediately vs. 1st)	After 2nd post-dilation (mm)	*p* value (1st vs. 2nd)
D-P group (n = 4)	28.0	28.08 ± 0.10	0.1586	29.35 ± 0.10	< **0.0001**	29.65 ± 0.10	**0.0054**
PAT group (n = 3)	28.0	28.13 ± 0.12	0.0750	28.73 ± 0.12**	**0.0036**	28.87 ± 0.12***	0.2262

D, distal; P, proximal; n, number of repetitions for the bench test; PAT, proximal anchoring technique. A *p*-value of statistical significance is shown in bold. **p < 0.01, and ***p < 0.001 compare with D-P group. All data are expressed as mean ± SD.

**Table 2.** **Change in stent length during each step of the procedure**.

	After 1st dilation stent lengthened toward the proximal (mm)	*p* value (D-P vs. PAT)	After 2nd dilation stent lengthened toward the proximal (mm)	*p* value (D-P vs. PAT)
D-P group (n = 4)	1.28 ± 0.19	**0.0018**	0.30 ± 0.20	0.2534
PAT group (n = 3)	0.60 ± 0.00***		0.13 ± 0.12	

D, distal; P, proximal; n, number of repetitions for the bench test. A *p*-value of statistical significance is shown in bold. ***p < 0.001 compare with D-P group. Data were all expressed as mean ± SD.

## 4. Clinical Serial PAT Cases 

**Fig. 4.**
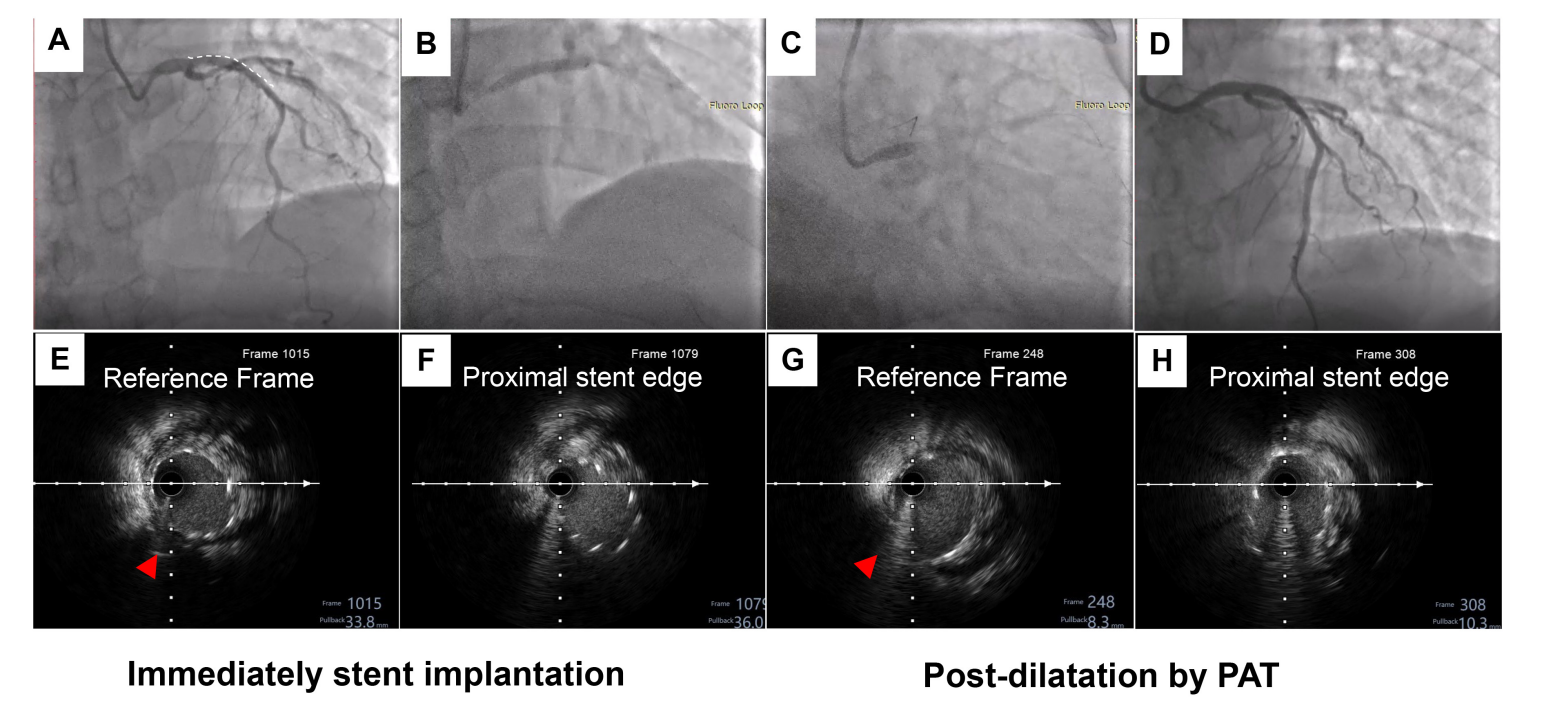
**The first representative case of PAT**. (A) Angiography showed a moderate ostial LM stenotic lesion and diffuse moderate stenosis lesions of the LAD. (B) Two drug eluting stents, a 2.75 × 23 mm and a 4.0 × 28 mm, were sequentially implanted, and the proximal stent edge was located at the LM ostium. (C) PAT was performed with a 4.0 × 12 mm non-compliant balloon. (D) The final results of angiography based on the IVUS-guided optimal procedure. (E,F) IVUS performed immediately after stent implantation showed that the length was 2.13 mm from the proximal stent edge to the ostium of the LM. (G,H) After PAT, the length was unchanged. Red arrows indicate a measurement marker of the ostial LM. IVUS, Intravenous ultrasound; LM, left main; LAD, left anterior descending. The white dashed line represents the length of the lesion.

We describe clinical cases performed with precise stent location and followed by PAT or conventional techniques distal to proximal post-dilation. The first patient presented with left main (LM) moderate stenotic lesions and diffuse moderate stenotic lesions of the left anterior descending (LAD) artery. After pre-dilatation, two DES were sequentially implanted at the middle of the LAD to the LM, and the proximal edge of stent was precisely located at the ostium of aorta-to-coronary which was achieved by the flowing wire technique (Fig. 4A,B). Then, sequential post-dilation was performed with PAT using a 4.0 × 8 mm non-compliant balloon post-dilatated at 16 atm and followed by a 2.75 × 15 mm non-compliant balloon. Intravascular ultrasound (IVUS) (Boston Scientific, Marlborough, MA, USA) was used to check for stent deformation during each step of the procedure. Compared with immediate stent implantation, the stent length measured by IVUS showed that there was no proximal longitudinal extension after PAT (Fig. 4E–H). In addition, there was no longitudinal geographic miss after PAT and no stent protrusion into the aorta. The procedure was then performed as per standard practice with an excellent final result (Fig. 4C,D).

**Fig. 5.**
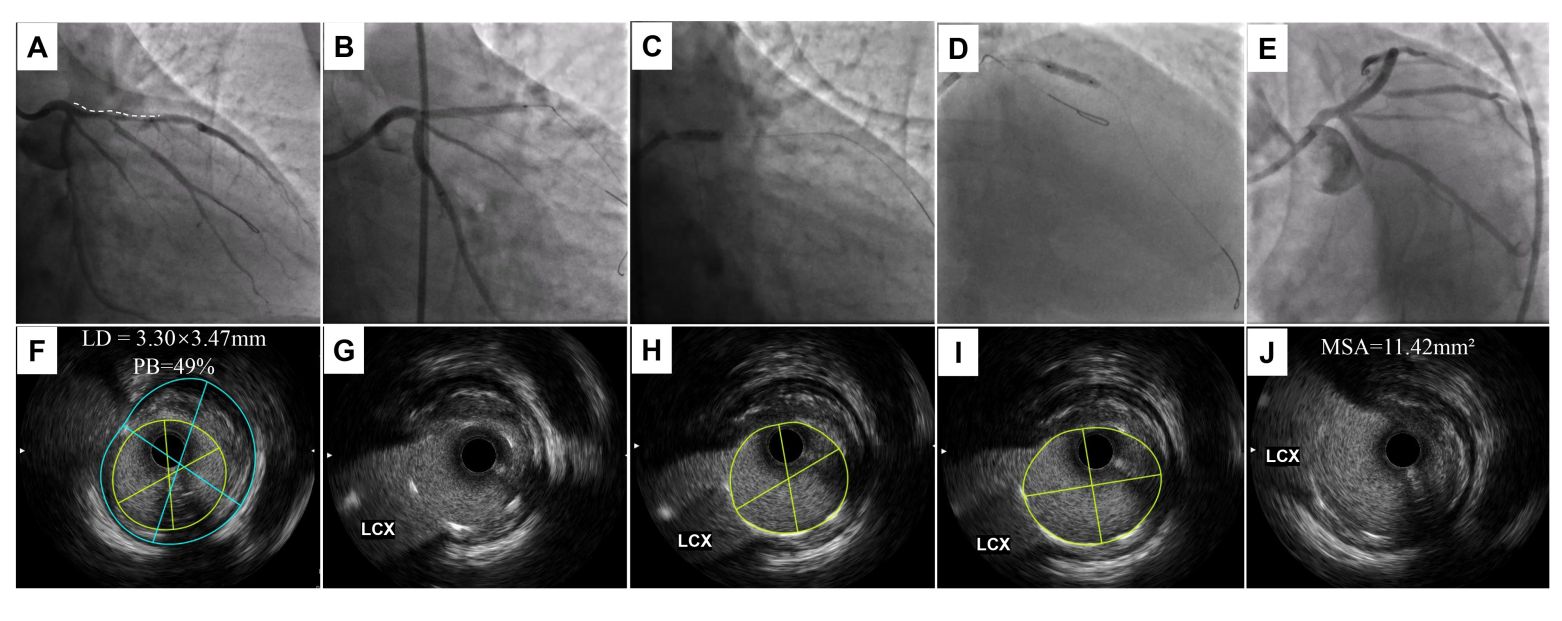
**The second representative case of PAT**. (A) Angiography showed a severe stenotic lesion from the ostium to the middle of the LAD. (B) A 3.0 × 33 mm DES was implanted and precisely located at the LAD ostium. (C,D) Post-dilation was performed with a 4.0 × 8 mm non-compliant balloon at 16 atm with PAT, followed by 3.0 × 15 mm, 3.5 × 15 mm and 3.75 × 9 mm non-compliant balloons from the distal to proximal LAD. (E) The final results of angiography based on the IVUS-guided optimal procedure. (F) The baseline IVUS check showed the PB and LD at the ostial LAD. (G–J) The IVUS check after post-dilation by PAT (G) and others with a non-compliant balloon, and (J) the final stent proximal edge showed no change compared to immediately following stent implantation. LD, lumen diameter; MSA, minimal stent area; PB, plaque burden. The white dashed line represents the length of the lesion.

The coronary angiography of another patient who was undergoing PAT showed an isolated lesion at the ostium of the LAD, with plaques extending to the mid-segment of the LAD (Fig. 5A). Since IVUS showed no significant stenosis at the root of the LM or ostium of the LCX, precise stent positioning was required (Medina 0,1,0). A 3.0 × 33 mm DES was placed in the proximal-to-mid LAD, with the proximal struts of the stent precisely positioned at the ostium of the LAD (Fig. 5B). Subsequently, the PAT was performed with a 4.0 × 8 mm post-dilation balloon at 16 atm in the proximal segment of the stent (Fig. 5C–E). The IVUS evaluation revealed that, compared to immediate stent implantation, the proximal stent edge did not extend into the POC region or affect the ostium of the LCX after PAT (Fig. 5F–J).

**Fig. 6.**
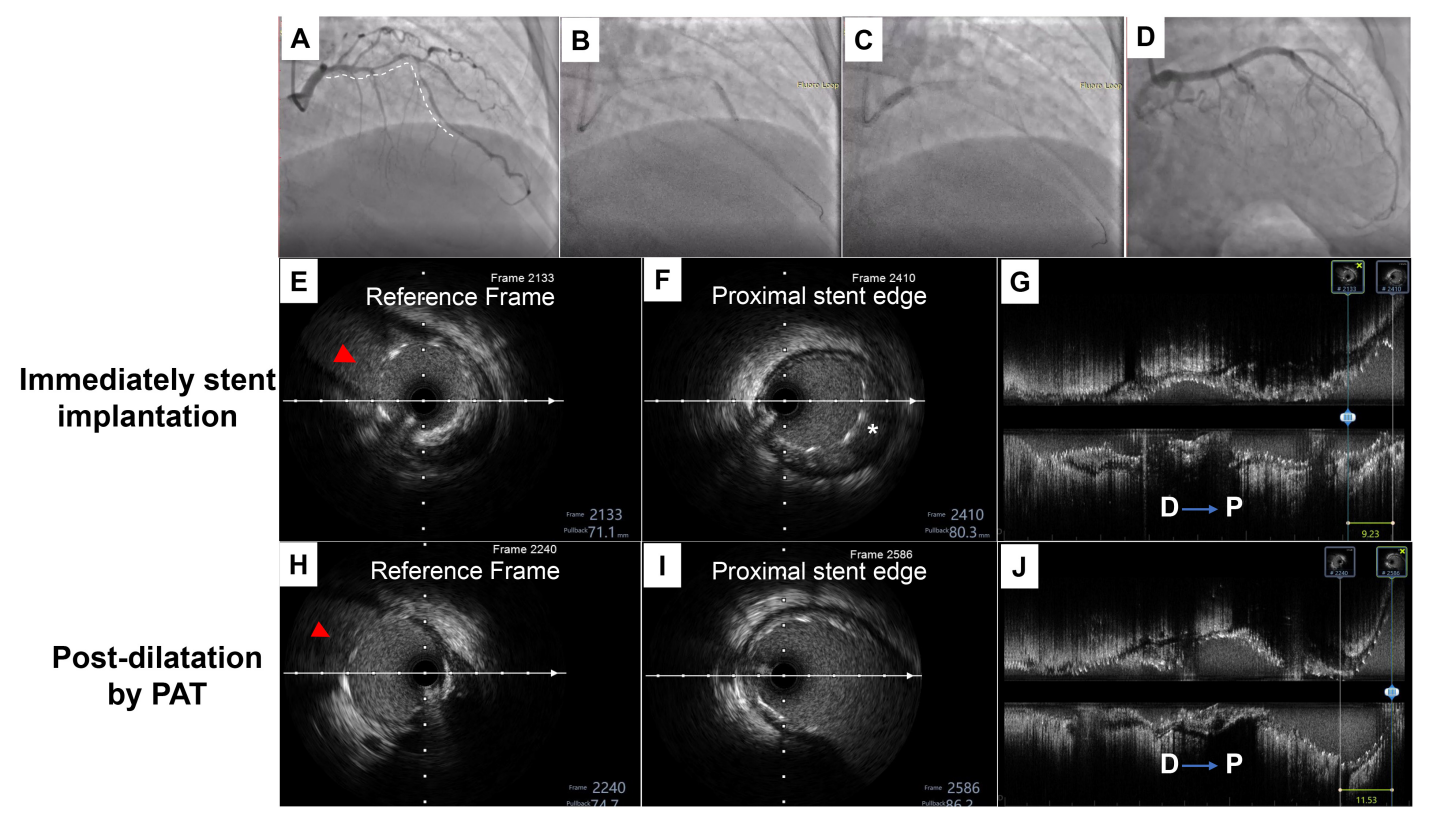
**A representative case of conventional post-dilation distal to proximal**. (A) Angiography showed diffuse severe stenosis from the proximal to the middle of left anterior descending (LAD). (B) Three drug eluting stents, a 2.25 × 24 mm, a 3.0 × 29 mm and a 4.0 × 18 mm, were sequentially implanted at the middle of the LAD to the LM. (C) Conventional post-dilation from the distal to proximal direction was performed with a 2.5 × 15 mm, a 3.0 × 12 mm, a 3.5 × 12 mm, a 4.0 × 8 mm and a 4.5 × 8 mm non-compliant balloons. (D) The final results of angiography based on the IVUS-guided optimal procedure. (E–G) IVUS check after immediate stent implantation showed that the length is 9.23 mm from the proximal stent edge to the carina of the LAD and LCX. (H–J) After conventional post-dilation, the length was significantly elongated to 11.53 mm, the Δ change in length was 2.3 mm. Red arrows indicate a measurement marker of the carina of the LAD and LCX. White asterisks indicate that the proximal stent edge is malpositioned. The white dashed line represents the length of the lesion.

The third patient presented with diffuse severe stenosis from the proximal to mid LAD. The IVUS check confirmed plaque extension into the polygon of confluence (POC). In order to avoid missing the geography, we decided to crossover the stent from the LAD to LM. A pre-dilation balloon was used to treat the LAD lesions, which was followed by three DES implanted at the mid LAD to LM. Sequential post-dilation was then performed using conventional techniques from the distal to proximal direction (Fig. 6A–D). Longitudinal stent deformation during each step of the procedure was checked by IVUS. Compared with immediate stent implantation (Fig. 6H–J), IVUS showed that the length was longitudinally extended 2.3 mm to the proximal portion of the LAD after post-dilation (Fig. 6E–G).

## 5. Discussion

In this study, we have described the PAT, a novel proximal post-dilation technique that aims at preventing the LSD. By utilizing bench modeling and the analysis of clinical cases, we provide novel insights into stent deformation related to post-dilation direction. We found that the D-P group showed a significant elongation in stent longitudinal length compared with the PAT group in the bench testing model. In addition, we found the PAT would help minimal proximal elongation and not affect the precise ostium stent implantation by analysis derived from clinical cases.

LSD has been described as longitudinal elongation or shortening of the stent in the longitudinal axis [7, 16]. Algowhary and Abdelmegid [11] analyzed 102 consecutively deployed stents by IVUS and found that 67.6% of implanted stents became elongated compared with the box-stated length. We know that even small changes in stent length can cause severe problems, especially in stents used for the treatment of aorta-ostial or some bifurcation lesions (Medina 0,1,0 and 0,1,1 and 0,0,1). Although there are several methods to help accurately locate the stent edge, such as multiple view angiography, the Szabo technique and the floating wire, the precise proximal location of the stent can be changed due to post-dilation ballooning [17].

Previous studies demonstrated that withdrawal of the device through deployed stents and extrinsic compression are involved in the longitudinal stent elongation [8, 18]. Matsuda *et al*. [19] retrospectively evaluated the effectiveness of post-dilation balloons on LSD in 103 stents using OCT, and found that stent elongation length was significantly longer in the mal-apposed group than in the well-apposed group. Most coronary arteries are anatomically shaped, however, the size of the stent often selected depends on the diameter of the distal reference vessel, which leads to mal-apposition of the proximal edge of the stent immediately upon placement. Stents performed with the proximal optimization technique (POT) increase the frictional force between the stent struts and the vessel wall to prevent the stent from elongating into the proximal direction. We hypothesize that PAT had a positive impact on preventing proximal stent elongation which may underlie the similar mechanisms with POT.

PAT has different indications than with POT. The latter is considered to be mandatory in true bifurcation lesions (Medina 1,1,0 and 1,1,1) which need to be performed with crossover or two-stent implantation techniques. However, the PAT is indicated for ostial or some bifurcation lesions (Medina 0,1,0 and 0,0,1) which always need precise stent placement. Second, the POT aims to complete circular correction of expected proximal strut mal-apposition [20, 21], to facilitate stent apposition in the proximal MB, to optimize strut clearance across the jailed side branch ostium, and to correct instrumentation-related stent deformation. The PAT is a novel post-dilation strategy with a different post-dilation direction using reference size non-compliant balloons according to the proximal lumen to avoid the LSD. Third, Toth *et al*. [6] recently found that POT with excessive overexpansion would cause significant proximal stent longitudinal elongation. They assumed that the overexpansion with tight ‘balloon-to-strut’ interaction results in longitudinal stretching of crowns and connectors, and that the POT balloon slides backwards during opening which pulls stent struts back from the distal portion of the ostium of the main branch. In consideration of these differences between POT and PAT, we advise that these two post-dilation methods should not be substituted for each other.

In addition, some studies showed that the post-dilation direction also impacts the stent longitudinal elongation, which is where the initial design of PAT originates. In a previous bench study, Sumi *et al*. [22] found that the distal to proximal post-balloon dilatation showed linear elongation during each step of the post-balloon dilatation. In contrast, during the proximal to distal post-balloon dilatation, the most significant change was observed in the first step of post-dilation and only slight changes were observed thereafter. Similar to these results, we found that the PAT group only had extended after the first post-dilation, but no further elongation thereafter, which is very crucial since these lesions need accurate location of the proximal stent edge.

There are some important details related to the use of PAT. First, the PAT balloon should be positioned with its proximal marker accurately located at the proximal end of the stent. Second, only non-compliant balloons (diameter of balloon was 1~1.2:1 ratio to the proximal reference vessel) are used for the PAT, to ensure accurate strut apposition and avoid excess overexpansion (always smaller than or equal to 16 atm). The balloons should always be completely deflated first and removed under visual control in order to avoid any stent entrapment when pulling back the deflated balloon catheter. Potential proximal elongation should be considered before stent implantation. Since PAT has minimal effect on stent elongation, the stent should be positioned slightly away from the ostium of the lesion.

## 6. Limitations

Despite the promising findings from our bench testing and initial clinical experience, this study has several limitations that should be acknowledged. First, the bench test was conducted using a single stent platform. Different stent platforms vary in their cell design, connector type, and material composition, which may influence their susceptibility to longitudinal deformation. Therefore, the generalizability of our results to other contemporary stent platforms requires further validation. Second, the use of a static silicone phantom, while valuable for a controlled initial assessment, cannot fully replicate the complex biomechanical environment of a human coronary artery, including pulsatile flow, vascular tortuosity, and the dynamic interaction with atherosclerotic plaques. Future studies utilizing more sophisticated biomechanical models or animal studies could provide further insights. Third, the clinical evidence is based on a small number of illustrative cases from a single center without a control group for direct comparison. The limited number of patients precludes any definitive conclusions regarding the clinical efficacy and safety of PAT. Furthermore, we did not provide long-term follow-up data to assess the durability of the technique and its impact on clinical endpoints such as target lesion revascularization or stent thrombosis. Finally, the operators in both the bench and clinical studies were not blinded to the post-dilation strategy, which could introduce observation bias. A larger-scale, prospective, randomized controlled trial is warranted to confirm the benefits of PAT over conventional techniques and to establish its role in contemporary PCI practice.

## 7. Conclusions 

We present a novel technique—PAT—to assist in preventing LSD. It has the potential to optimize current practices with stent implantation of ostial lesions and partial bifurcation lesions (Medina 0,1,0 and 0,1,1 and 0,0,1), which need massive over-dilation and accurate stent placement. In this study, only bench testing and clinical case series were reported. The long-term outcomes require further investigation in a prospectively designed study.

## Availability of Data and Materials

The datasets used and analyzed during the current study are available from the corresponding author on reasonable request.

## Supplementary Material

Supplementary material associated with this article can be found, in the online version, at https://doi.org/10.31083/RCM44250.
