# Supplementary figures and images for: Proximal Anchoring Technique: A Novel Technique to Prevent Coronary Stent Longitudinal Deformation
Source: Rev Cardiovasc Med. 2026 Feb 10;27(2):44250. doi: 10.31083/RCM44250 (PMC12959999; doi:10.31083/RCM44250)

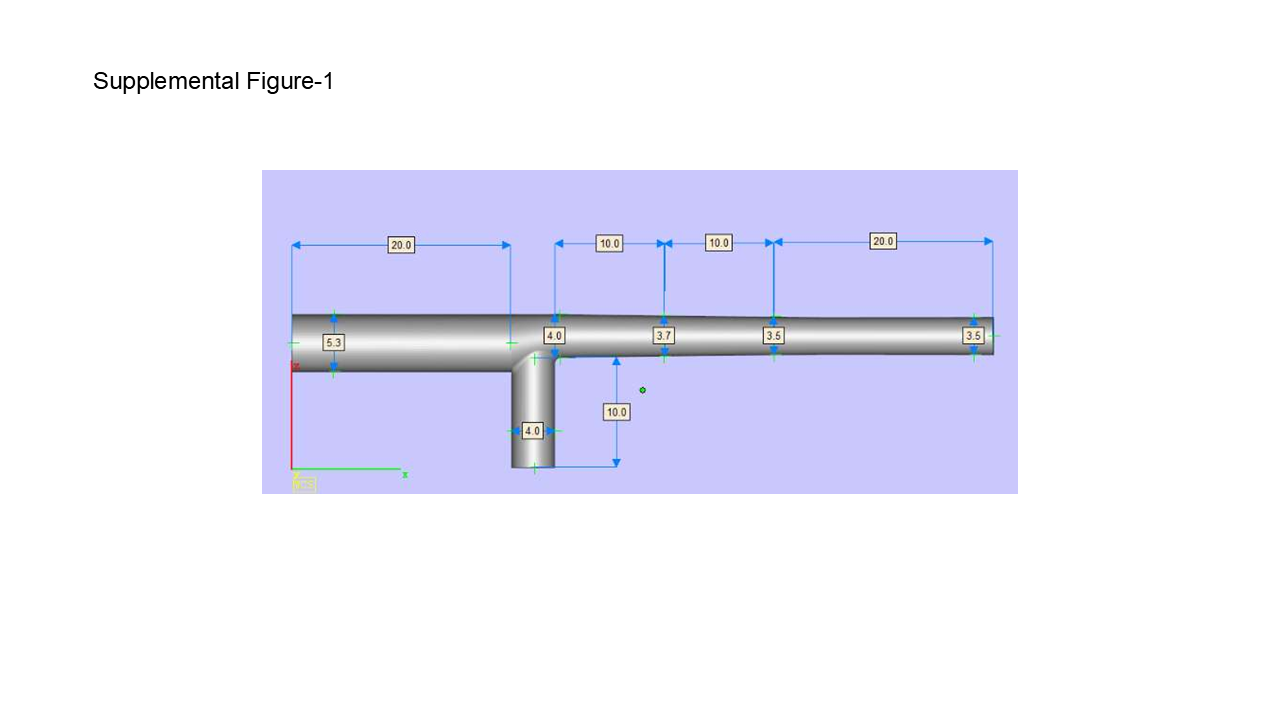

Supplement: Supplementary file 1 [file 2153-8174-27-2-44250-s1.zip › Supplementary Fig. 1.TIF]

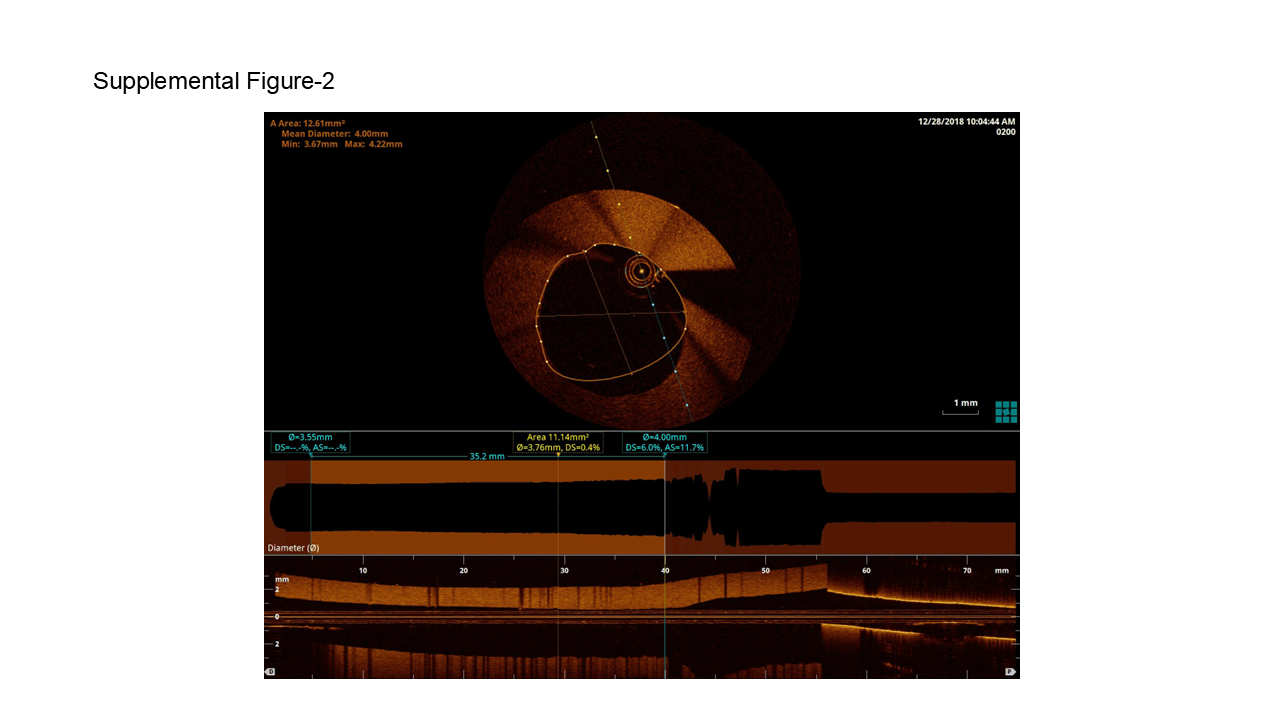

Supplement: Supplementary file 1 [file 2153-8174-27-2-44250-s1.zip › Supplementary Fig. 2.TIF]
